# ESPO & Butler-Williams Symposium: Career Development to Promote Innovations From Bench to Bedside

**DOI:** 10.1093/geroni/igaf122.1096

**Published:** 2025-12-31

**Authors:** Mirella Diaz-Santos, Brianna Morgan, Ramesh Vemuri

## Abstract

The NIA’s Butler-Williams Scholars Program and GSA’s ESPO section are united in providing career development opportunities to promote leadership and innovation. This year’s theme challenges our established and emerging scholars to embrace innovations addressing motivators, barriers, and facilitators to translational research with older adults from bench to bedside. Despite representing about 17% of the U.S. population, older adults remain under-represented in most clinical research and trials. This suggests that the current treatments are not fully generalizable to aging populations, and addressing this scientific gap is urgently warranted. GSA’s early career professionals and alumni of the prestigious NIA Butler-Williams Scholars Program will address these gaps. In a qualitative study, Dr. Maira Castañeda-Avila will present patient-level and healthcare-level barriers and facilitators in recruiting older adults and individuals with multiple chronic conditions. Dr. Miriam J Rodriguez will extrapolate factors contributing to dementia caregiving experiences among informal caregivers and their impact on clinical trial participation, specifically in a caregiver-targeted intervention. Dr. Mirella Díaz-Santos will present the findings of a primary care champion intervention focused on engaging family medicine providers to recruit older adults and administer a dementia screening toolkit. The featured talks will engage us in expanding our collective innovations in recruitment to advance patient-centered treatments for aging populations. Dr. Benjamin F Miller, PhD will discuss his career journey in translating basic biology of aging into treatments to increase human healthspan, with a focus on proteostatic mechanisms in mitochondria to increase stress resistance for slowing aging.

